# Stochastic multicellular modeling of x-ray irradiation, DNA damage induction, DNA free-end misrejoining and cell death

**DOI:** 10.1038/s41598-019-54941-1

**Published:** 2019-12-11

**Authors:** Jake C. Forster, Michael J. J. Douglass, Wendy M. Phillips, Eva Bezak

**Affiliations:** 1 Department of Nuclear Medicine, South Australia Medical Imaging, The Queen Elizabeth Hospital, Woodville South, SA 5011 Australia; 2 Department of Physics, University of Adelaide, Adelaide, SA 5005 Australia; 3 Department of Medical Physics, Royal Adelaide Hospital, Adelaide, SA 5000 Australia; 4 Cancer Research Institute and School of Health Sciences, University of South Australia, Adelaide, SA 5001 Australia

**Keywords:** Computational biophysics, Cancer therapy, Head and neck cancer, Computational models, Biological physics

## Abstract

The repair or misrepair of DNA double-strand breaks (DSBs) largely determines whether a cell will survive radiation insult or die. A new computational model of multicellular, track structure-based and pO_2_-dependent radiation-induced cell death was developed and used to investigate the contribution to cell killing by the mechanism of DNA free-end misrejoining for low-LET radiation. A simulated tumor of 1224 squamous cells was irradiated with 6 MV x-rays using the Monte Carlo toolkit Geant4 with low-energy Geant4-DNA physics and chemistry modules up to a uniform dose of 1 Gy. DNA damage including DSBs were simulated from ionizations, excitations and hydroxyl radical interactions along track segments through cell nuclei, with a higher cellular pO_2_ enhancing the conversion of DNA radicals to strand breaks. DNA free-ends produced by complex DSBs (cDSBs) were able to misrejoin and produce exchange-type chromosome aberrations, some of which were asymmetric and lethal. A sensitivity analysis was performed and conditions of full oxia and anoxia were simulated. The linear component of cell killing from misrejoining was consistently small compared to values in the literature for the linear component of cell killing for head and neck squamous cell carcinoma (HNSCC). This indicated that misrejoinings involving DSBs from the same x-ray (including all associated secondary electrons) were rare and that other mechanisms (e.g. unrejoined ends) may be important. Ignoring the contribution by the indirect effect toward DNA damage caused the DSB yield to drop to a third of its original value and the cDSB yield to drop to a tenth of its original value. Track structure-based cell killing was simulated in all 135306 viable cells of a 1 mm^3^ hypoxic HNSCC tumor for a uniform dose of 1 Gy.

## Introduction

One of the tasks of radiobiological modeling is to predict whether a cell that received ionizing radiation will ultimately die from the initial radiation-induced DNA damage, broadly consisting of DNA double-strand breaks (DSBs), single-strand breaks, modified bases and modified sugars. Cell death (defined by the loss of replicative potential) after irradiation mainly occurs in the form of mitotic catastrophe, meaning it results from, or follows, aberrant mitosis^1–3^. This occurs when a chromosome aberration (CA) is present that is “asymmetric”, preventing a large amount of the genetic material from being replicated.

This paper presents a new computational model of CA production and cell death from ionizing radiation. First, some background is provided on the mechanism of CA production by DSB misrepair and the relationship between CAs and cell death. Other computational models of radiation-induced CAs and cell death in the literature are reviewed and a gap is identified that will be addressed by the current model.

## Mechanism of CA Production by DSB Misrepair

**Figure 1:**
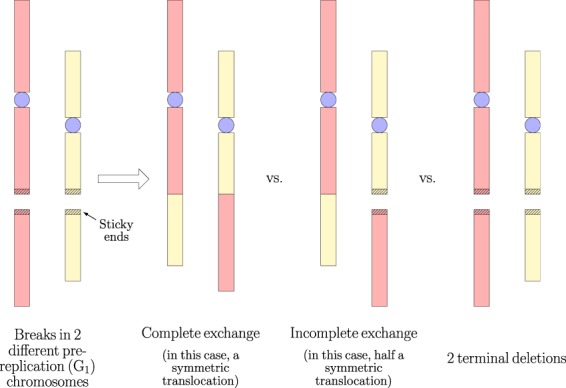
DSB misrepair can be in the form of complete exchanges, incomplete exchanges and terminal deletions.

CAs are formed by the misrepair of DSBs. Each DSB causes a break in a chromosome, producing two DNA free-ends (a.k.a. “sticky” ends). Unrelated (incongruent) DNA free-ends produced by different DSBs can be misrejoined (illegitimate/incorrect reunion or exchange), resulting in an exchange-type CA^4–7^. A DSB can also have both ends left unrejoined, resulting in a terminal deletion, or have one end misrejoined and the other end left unrejoined (an incomplete exchange) (Fig. 1)^8^.

**Figure 2:**
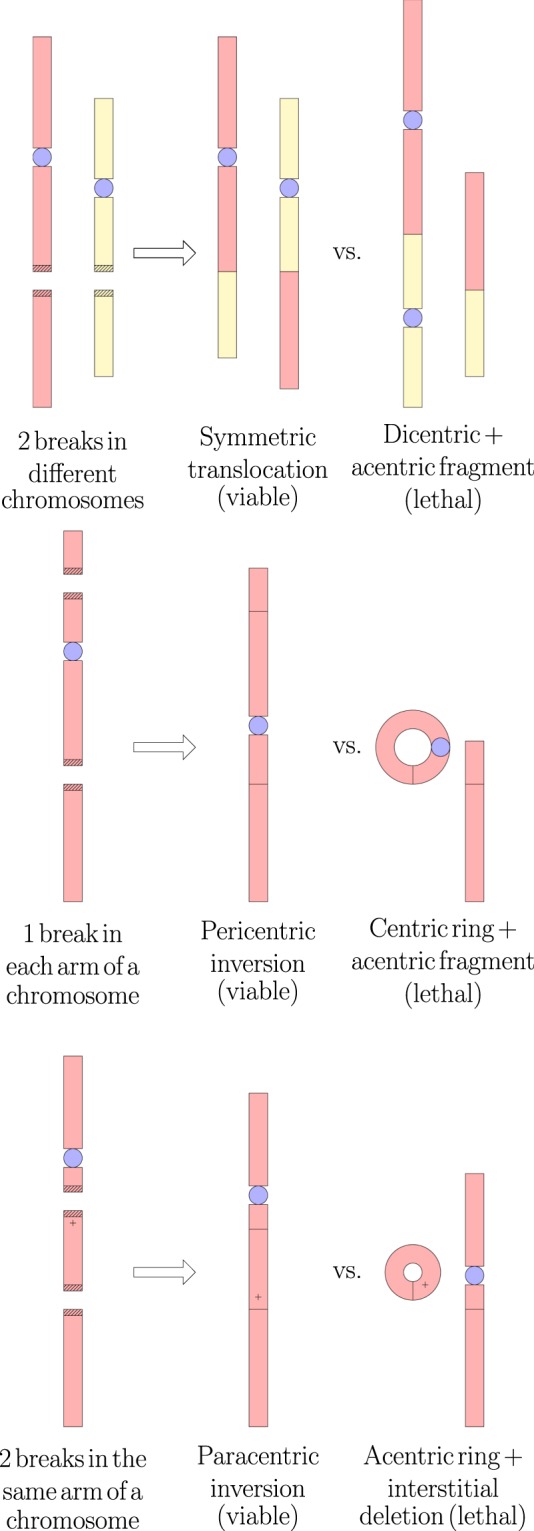
Simple exchange-type CAs. Complete exchanges are shown.

“Simple” exchange-type CAs are those that are possible with just two DSBs (Fig. 2)^9^. If the two DSBs are on different chromosomes, a symmetric translocation or a dicentric (plus an acentric fragment) can form. Two DSBs on opposite arms of a chromosome can give rise to a centric ring (plus an acentric fragment) or a pericentric inversion. If the two DSBs are on the same arm, an acentric ring (plus an interstitial deletion) or a paracentric inversion can occur. A plethora of “complex” exchange-type CAs are possible with three or more DSBs on two or more chromosomes.

Of the simple exchange-type CAs, dicentrics and rings (centric and acentric) are asymmetric. Since they cause a large amount of the genetic material to be lost at the next mitosis, they are unstable (lethal). On the other hand, symmetric translocations and inversions (pericentric and paracentric) are symmetric and stable (non-lethal). Large deletions due to unrejoined ends are also lethal.

The main repair pathway for DSBs in all cell cycle phases for mammalian cells is canonical-non-homologous end-joining (c-NHEJ)^6,10–12^. There are two types of c-NHEJ in G_0_/G_1_: one fast (2–4 hrs) and one slow (>8 hrs). The fast one is resection-independent and the slow one is resection-dependent (and Artemis-dependent). The slow one is more error prone. For example, in G_1_ human cells, resection-independent and resection-dependent c-NHEJ repaired 80% and 20% of DSBs, respectively, but each was responsible for half of the misrejoinings after a 7 Gy dose of 90 kV x-rays^13^. In G_2_, resection-independent c-NHEJ is active but not resection-dependent c-NHEJ. The slow repair pathway in G_2_ is homologous recombination (HR), which is error-free. HR is also active in S. There is also the alternative-NHEJ (alt-NHEJ) pathway, which involves enzymatically-driven resection. This is slow and error-prone (similar to resection-dependent c-NHEJ), but does not seem to contribute to repair in G_0_/G_1_-phase human cells (though it does appreciably in mice)^10^.

Since DNA free-ends must spatio-temporally co-localize to either rejoin or misrejoin, it appears that the slowness of resection-dependent c-NHEJ and alt-NHEJ is what makes them more error-prone than resection-independent c-NHEJ, which is fast: the DNA free-ends have more time to move away from their initial location (possibly with Brownian motion^14^) and meet incongruent DNA free-ends. It follows that a misrejoining is more likely to occur if the DSBs are initially closer together.

The complexity of a DSB appears to affect the choice of repair pathway. Complex DSBs are repaired by slow pathways and simple DSBs by fast pathways^15,16^, though it is unclear why this is the case^11,17^. Accordingly, DNA free-ends from complex DSBs are more likely to be misrejoined.

The average number of CAs per cell, *N*_*ca*_, is typically linear-quadratic with dose^4,7^:

1 $${N}_{ca}={\alpha }_{ca}D+{\beta }_{ca}{D}^{2}$$

The linear component receives contributions from misrejoinings involving DSBs produced by the same primary (e.g. the same x-ray, including all associated secondary electrons) and from terminal deletions. The quadratic component corresponds to misrejoinings involving DSBs produced by two different primaries (thus it depends on dose rate and fractionation).

Let *f* ≤ 1 be the fraction of lethal CAs, so the average number of lethal CAs per cell is:

2 $${N}_{lca}=f{N}_{ca}$$

If the fraction of lethal CAs is independent of dose and lethal CAs are Poisson distributed in cells (the latter assumption may become less valid with increasing dose^18^), the surviving fraction from lethal CAs is:

3 $${S}_{lca}={e}^{-{N}_{lca}}$$

4 $$\,=\,{e}^{-f({\alpha }_{ca}D+{\beta }_{ca}{D}^{2})}$$

5 $$\,=\,{e}^{-{\alpha }_{{\rm{killing}}(lca)}D-{\beta }_{{\rm{killing}}(lca)}{D}^{2}}$$

Thus the surviving fraction inherits the linear-quadratic dependence from the CA yield^2^. The linear-quadratic model generally provides a good fit to experimental data from a clonogenic cell survival assay for doses up to about 10 Gy per fraction^19^.

It is important to emphasise that *S*_*lca*_ is the contribution to the surviving fraction from the mechanism of lethal CA-driven mitotic catastrophe. While this appears to be the main mechanism of radiation-induced cell death for mammalian cells^1–3^, there is evidence for additional sources of cell death. Sensitivity to apoptosis is often reduced in cancer compared to normal tissue^20^, but apoptosis may be an important contributor to radiosensitvity in some tumors^1^. Apoptosis can occur in response to DNA damage^21,22^. In addition, damage to mitochondria (which can be enhanced by gold nanoparticles) can trigger cell death by apoptosis^23^. There are also non-targeted effects such as bystander effects, wherein irradiated cells release harmful signals that are received by other cells^24^. In the approximation that the different mechanisms contribute independently (i.e. no synergistic or antagonistic effects), the overall surviving fraction is given by the product of all the contributions:

6 $$S={S}_{lca}{S}_{2}{S}_{3}\mathrm{..}.$$

Macroscopic cell death is the convolution of many microscopic processes. By modeling a single microscopic process such as the production of CAs, its contribution to cell death can be investigated in isolation.

## Computational Models of Radiation-Induced CAs and Cell Death

Deterministic models of DSB repair and misrepair in the literature include^25–27^. There are also stochastic models, which stochastically generate a spatial distribution of DSBs in the nucleus (e.g. from Monte Carlo track structure) and then simulate the fate of each individual DSB using stochastic methods. The model in the current work is stochastic. Examples of stochastic models in the literature include the model by Henthorn *et al*.^28^, Friedland and Kundrát’s model^29,30^, “BIophysical ANalysis of Cell death and chromosome Aberrations” (BIANCA)^31–33^ and Brenner’s model^34^.

Brenner’s model simulated track segments using the Monte Carlo code PROTON. A track was generated and then a cell nucleus was randomly superimposed over the track to score a segment of the track in the nucleus. This was repeated for a number of tracks (a certain number of tracks were expected to pass through the nucleus for a given dose), then it was assumed 1 in 1500 ionizations produced a DSB. The model simulated time-dependent diffusion and distant-dependent interaction of the DSBs to form exchange-type CAs, in competition with faithful DSB repair.

The BIANCA model simulated cluster lesions (CLs), which were effectively DSBs whose free-ends were able to misrejoin, in a single cell nucleus. For sparsely-ionizing photons, the CLs were placed at random in the nucleus. For protons and alphas, there was an expected number of nucleus traversals (based on dose) and an expected number of CLs from each traversal (based on LET). Line segments representing traversals were randomly simulated through the nucleus and CLs were randomly distributed along the line segments. For higher LETs, some of the CLs were placed radially with respect to the line segments to account for high-energy secondary electrons. Proximity-based misrejoining of the DNA free-ends from CLs was simulated. The nucleus contained interphase chromosome territories and chromosome-arm domains, making it possible to determine the types of CAs produced (e.g. dicentrics, centric rings, etc.).

Friedland and Kundrát’s model simulated Monte Carlo tracks through a single cell nucleus using the code PARTRAC (PARticle TRACks) and superimposed the tracks with a multiscale DNA target model to generate DNA damage. Spatio-temporal motion of DNA free-ends was simulated in addition to mechanistic modeling of NHEJ to achieve DSB repair and misrepair. The types of CAs produced were identifiable due to modeling of chromosome territories in the nucleus. The model by Henthorn *et al*. also simulated DNA free-end motion with mechanistic modeling of c-NHEJ. The DSBs were generated from Monte Carlo tracks simulated in Geant4^35–37^ using Geant4-DNA^38–40^, using a spatial clustering approach. Chromosome territories were not modeled.

Compared with these other stochastic models, the current model has two unique properties: (i) Monte Carlo tracks were simulated through a multicellular tumor volume (as opposed to a single cell nucleus) and (ii) variable partial pressures of oxygen (pO_2_) were simulated in cells. A multicellular tumor of head and neck squamous cell carcinoma (HNSCC) was irradiated with tracks using the Monte Carlo toolkit Geant4 (version 10.4)^35–37^. The physics and chemistry modules of Geant4-DNA^38–40^ were used to accurately simulate low-energy physical interactions and the chemical tracks from water radiolysis. The tracks through cell nuclei were translated into spatial distributions of DNA damage, including DSBs, by spatially clustering the direct and indirect events into simulated DNA volumes. The DNA damage from the tracks was subject to the cellular pO_2_: a higher pO_2_ enhances the conversion of DNA radicals to strand breaks. Proximity-based DNA free-end misrejoining was simulated. Cell death was then predicted from the presence of lethal CAs.

The purpose of this work was two-fold. First, to present new stochastic modeling of radiation-induced CA production and cell killing, that befits a different purpose than other models in the literature. Namely, the simulation of a multicellular tumor and variable pO_2_ conditions makes this model uniquely apt to be used as part of a radiotherapy model, i.e., a model that predicts the outcome of a radiotherapy treatment to a tumor. Radiotherapy models often take the approach of simulating each individual cell in the tumor, but they typically simulate the radiation effect using the Linear-Quadratic equation or a variant thereof^41–53^. Greater model accuracy and utility may be achieved by instead simulating the radiation effect starting from Monte Carlo track structure. Radiotherapy models may play an important role in testing hypoxia-targeting treatments, radiotherapy planning and treatment individualization in the future. The second aim of this work was to use the model to investigate the contribution of DNA free-end misrejoining to CA production and cell death by low-LET radiation for HNSCC.

## Methods

### Model methods

**Figure 3:**
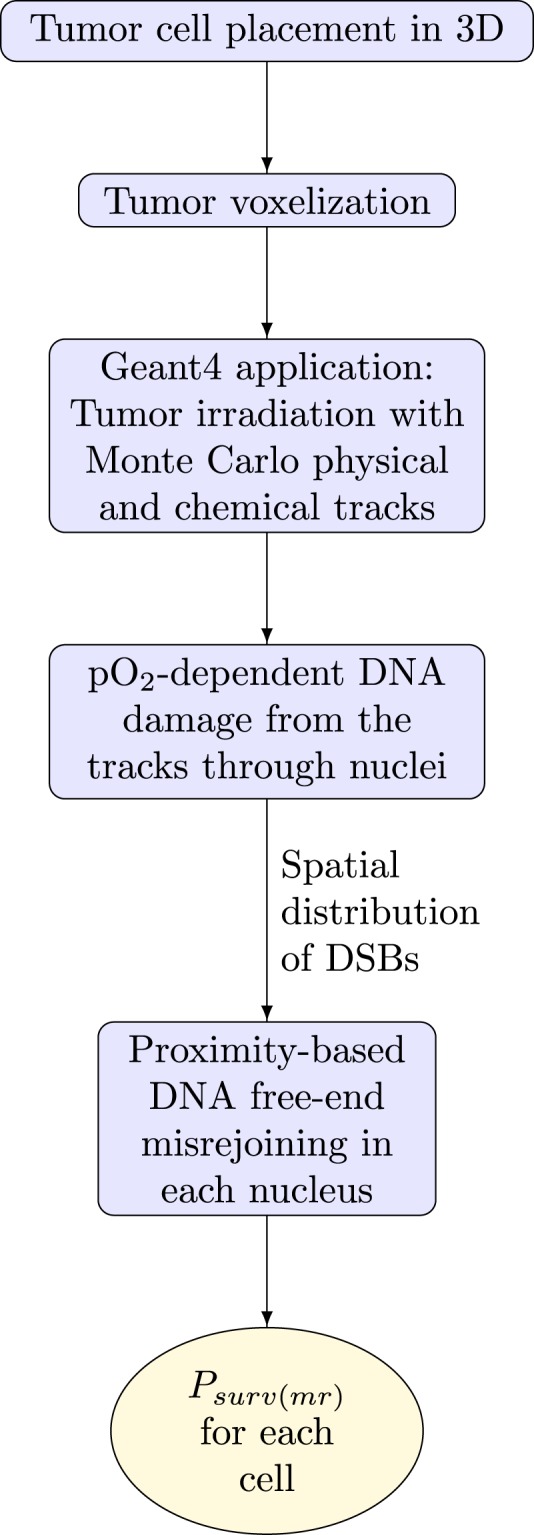
Basic flowchart of the model. ${P}_{surv(mr)}$ is the probability of cell survival by the mechanism of misrejoining.

A computational model was developed in-house to simulate a dose fraction to a multicellular tumor of HNSCC, followed by DNA damage induction, DNA free-end misrejoining and cell death from misrejoining. The flowchart in Fig. 3 summarises the components of the model, which are described in more detail below.

### Tumor cell placement in 3D

**Figure 4:**
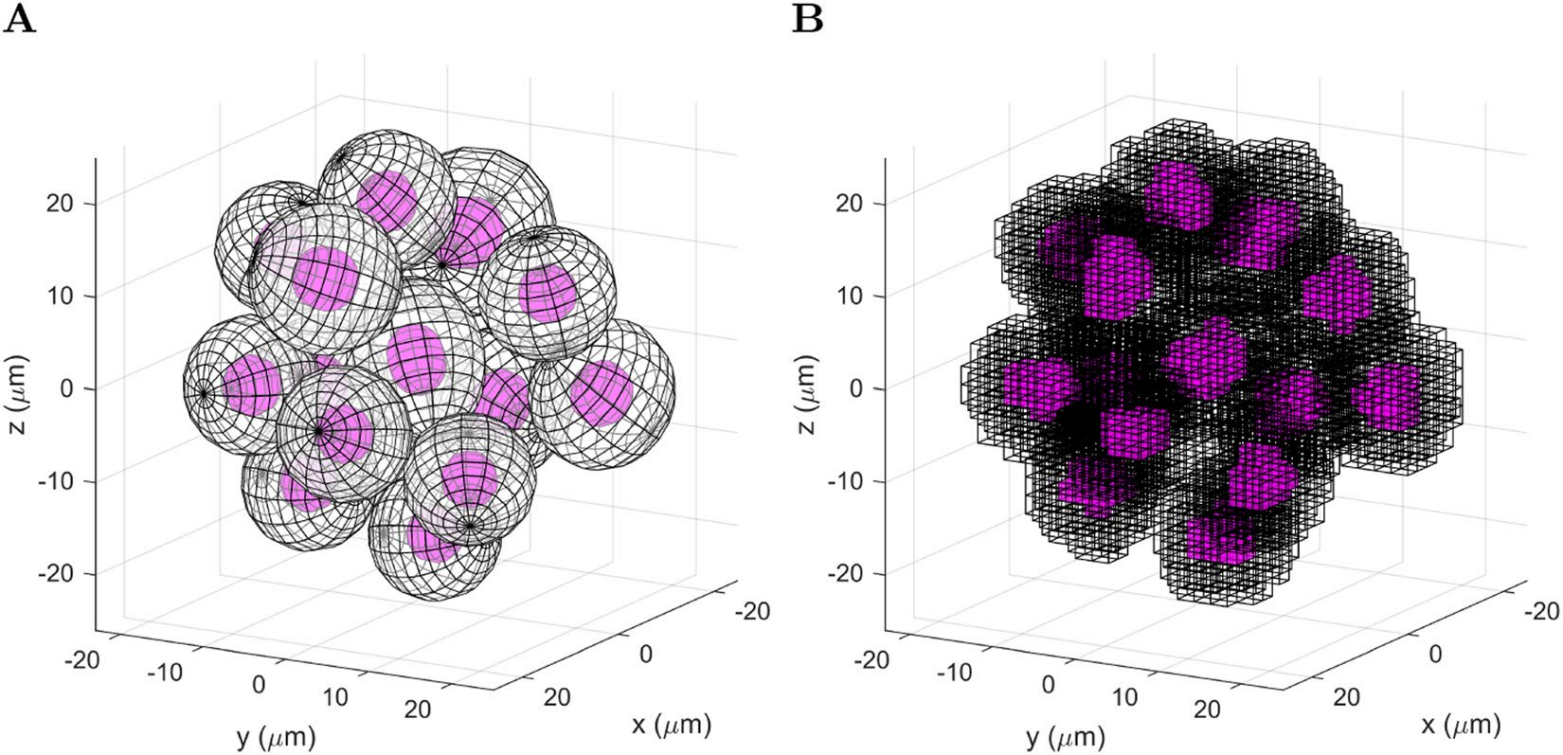
Example of a multicellular tumor with a small number of cells for demonstration. (**A**) Randomized ellipsoidal cells containing nuclei. (**B**) The corresponding voxel representation, showing nucleus and cytoplasm voxels.

A cubic volume of side length 0.2 mm was filled with 1224 non-overlapping cells (a density of 1.53 × 10^8^ cells/cm^3^, which is tumor-mimetic^54^) using a previously developed algorithm (Fig. 4A)^55,56^. The cells were ellipsoid-shaped with random orientations, major axis lengths from 14 to 20 *μ*m and remaining axis lengths of at least 14 *μ*m (cell volumes 1437 to 2053 *μ*m^3^), representative of FaDu HNSCC cells^57^. Cells contained a concentric nucleus that occupied 8% of the cellular volume (nuclear volumes 115 to 164 *μ*m^3^)^58^.

### Tumor voxelization

In order to import the multicellular tumor (1224 cells) into Geant4 (version 10.4)^35–37^, it was first voxelized. Voxels were cubic volumes of side length 2 *μ*m representing either nucleus, cytoplasm or intercellular material (Fig. 4B). Voxelization was performed using an algorithm written in Matlab (R2016b, MathWorks Inc., Natick, MA). The distance from the center of a voxel to the center of a cell was used to determine whether the center of the voxel was located within the nucleus or cytoplasm of the cell (accounting for the ellipsoid shape and the orientation). Voxels were assigned as nucleus or cytoplasm based on this criteria.

For the cubic volume of side length 0.2 mm, there were 100 × 100 × 100 = 10^6^ voxels. Two 3D arrays of size 100 × 100 × 100 called *voxelType* and *voxelCell* were introduced. The element $\overrightarrow{i}=(i,j,k)$ of these arrays (where *i*, *j* and *k* took integer values from 0 to 99) corresponded to the voxel whose center had spatial coordinates:

7 $$\overrightarrow{X}=-\,99+2\overrightarrow{i}(\mu {\rm{m}})$$

The *voxelType* array contained the flag identifying the voxel type (0 for intercellular, 1 for nucleus and 2 for cytoplasm) and *voxelType* contained the flag identifying the cell that the voxel belonged to (0 for intercellular voxels, 1–1224 for nucleus and cytoplasm voxels).

### Tumor irradiation with monte carlo track structure

The voxelized multicellular tumor (1224 cells) was imported into Geant4 as follows. A cubic volume (soon to accommodate the tumor) of side length 0.2 mm was placed in Geant4 and divided into 100 × 100 × 100 voxels using “nested parametersiation”. Nucleus, cytoplasm and intercellular materials were defined equivalent to “G4_WATER” (required to use Geant4-DNA^38–40^) with density 1 g/cm^3^. The voxels were then assigned to nucleus, cytoplasm or intercellular material using the *voxelType* array.

**Figure 5:**
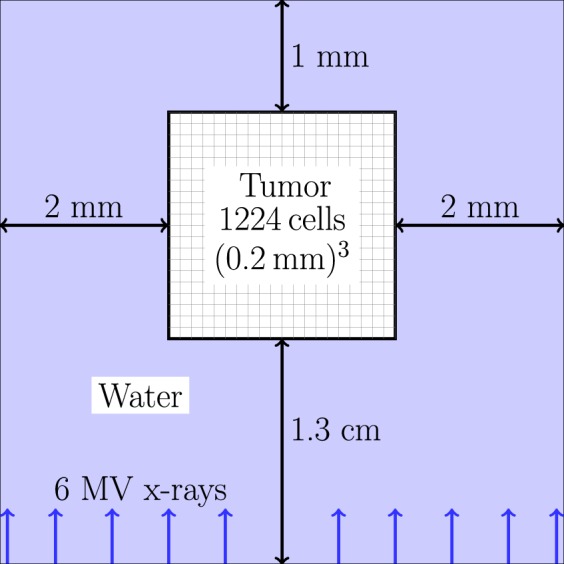
Irradiation set-up in Geant4. The voxelized multicellular tumor is encased in water.

X-rays with an energy distribution from a 6 MV linac model developed in the Philips Pinnacle treatment planning system were fired from a square 4.2 mm × 4.2 mm source with parallel beam, placed 1.3 cm from the tumor (Fig. 5). The tumor was encased in water to achieve a uniform dose to tumor (see Results; Section: sec:res-sens). Relative to the beam direction, there was 1.3 cm of water in front of the tumor to achieve electronic equilibrium in the tumor (for 6 MV x-rays the mean electron energy is ~2 MeV, which has a range of ~1 cm in liquid water using the continuous slowing down approximation^59^), 2 mm of water either side of the tumor for lateral scattering and 1 mm of water behind the tumor to provide back scatter medium (MV x-rays mostly scatter forward).

Geant4-DNA physics models, which accurately simulate physical interactions down to eV energies, were used to simulate electron interactions in the tumor for electron energies <1 MeV (the models were not defined ≥1 MeV). The Livermore physics list (which is low-energy but not as low as Geant4-DNA) was used in the tumor for electron energies ≥1 MeV and for photon and positron interactions (Table 1). Outside the tumor, Livermore physics was used exclusively (there was no need to simulate very low-energy physics outside of the tumor) (Table 2). For physical interactions in nucleus voxels that deposited ≥10.79 eV (the ionization energy for liquid water), the spatial coordinates of the interaction were recorded, along with the flag identifying the cell in which it took place (using *voxelCell*). The total energy deposited in each voxel was also recorded.

**Table 1:** Physics interactions simulated in the tumor.

Particle	Interaction	Energy range^a^	Model
e^−^	multiple scattering	≥1 MeV	Urban
	elastic scattering	(7.4 eV, 1 MeV)	DNA Champion
	ionization	≥1 MeV	Møller-Bhabha
		[10 keV, 1 MeV)	DNA Born
		[11 eV, 10 keV)	DNA Emfietzoglou
	excitation	[10 keV, 1 MeV)	DNA Born
		[8 eV, 10 keV)	DNA Emfietzoglou
	vibrational excitation	(7.4 eV, 100 eV)	DNA Sanche
	attachment	(7.4 eV, 11.2 eV)	DNA Melton
	electron solvation	≤7.4 eV	DNA one step thermalization
	bremsstrahlung		Livermore
*γ*	Compton		Livermore
	photoelectric effect		Livermore
	conversion		Livermore
	Rayleigh scattering		Livermore
e^+^	multiple scattering		Urban
	ionization		Møller-Bhabha
	bremsstrahlung		Seltzer-Berger
	annihilation		

^a^Energies relevant to this application with 6 MV x-rays. Active for all relevant energies if not specified.

**Table 2:** Physics interactions simulated outside of the tumor.

Particle	Interaction	Energy range^a^	Model
e^−^	multiple scattering		Urban
	ionization	≥100 keV	Møller-Bhabha
		<100 keV	Livermore
	bremsstrahlung		Livermore
*γ*^b^			
e^+b^			

^a^Energies relevant to this application with 6 MV x-rays. Active for all relevant energies if not specified. ^b^Same as in the tumor (Table 1).

**Figure 6:**
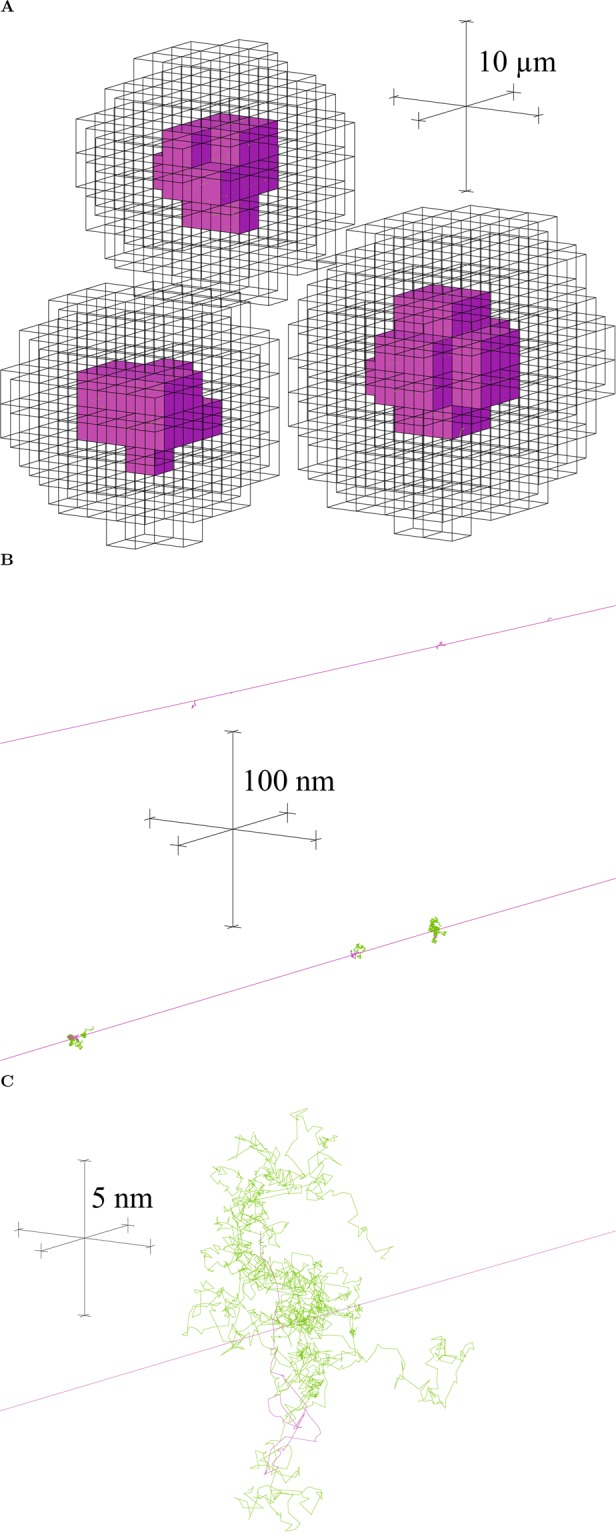
Visualization of a few cells irradiated with 1 MeV electrons. (**A**) The electron tracks are not drawn, but their passage through cell nuclei is dotted by ^•^OH tracks (green) (Geant4-DNA chemistry was only simulated in nucleus voxels). The three scale bars are mutually orthogonal and each 10 *μ*m long. (**B**) A closer view (100 nm scale bars) shows the ^•^OH tracks generated sporadically along the electron tracks (magenta). (**C**) A closer view again (5 nm scale bars) shows ^•^OH tracks in more detail, depicting 2.5 ns of diffusion by Brownian motion. Images were created using the Fukui Renderer DAWN (Drawer for Academic WritiNgs), version 3.90b, Satoshi Tanaka *et al*. https://geant4.kek.jp/tanaka/DAWN/About_DAWN.html.

Geant4-DNA chemistry was used to simulate water radiolysis following Geant4-DNA physics, but only inside nucleus voxels to save computation time (Fig. 6). ^•^OH was the only species that contributed to indirect DNA damage, neglecting the minor contributions from hydrogen atoms and solvated electrons^60,61^. It was assumed that ^•^OH molecules interacted (were scavenged) after 2.5 ns of diffusion. This corresponded to an ^•^OH scavenging capacity of 4 × 10^8^ s^−1^, and a root-mean-square displacement of 6.48 nm with the ^•^OH diffusion coefficient of 2.8 × 10^−9^ m^2^ s^−1^ used in Geant4-DNA chemistry, which are mimetic of the cellular environment^62–67^. When the virtual time of the chemistry simulation reached 2.5 ns, for each ^•^OH molecule inside a nucleus voxel, the spatial coordinates of the ^•^OH were recorded (taken to be the position of an ^•^OH interaction) along with the flag identifying the cell it was located in (using *voxelCell*). The chemistry simulation was ended after a virtual time of 2.5 ns to save computation time.

### DNA damage induction algorithm

A spatial distribution of DNA damage including DSBs, single-strand breaks, modified bases and modified sugars was generated in each tumor cell nucleus from the track structure, using an algorithm that was described previously^68^. Briefly, the algorithm spatially clustered the physical interactions that deposited ≥10.79 eV (direct events) and ^•^OH interactions (indirect events) in the nucleus into cylindrical volumes representing 10 base-pair segments of DNA. Direct events on DNA and its first hydration layer and indirect events on DNA produced DNA sugar radicals and base radicals, some of which were subsequently converted to strand breaks with pO_2_-dependent probabilities (the radiochemical oxygen effect; see^68^ for more detail on how this was modeled). Two or more strand breaks within 10 base-pairs constituted a DSB. If two DSBs were separated by less than 10 undamaged base-pairs, they were classified as the same DSB. Thus DSBs acquired a multiplicity. The DNA damage induction algorithm was compared with experimental data and other theoretical codes in^68^. Only the spatial distributions of DSBs were used hence.

Note that the chemical stage of the Geant4-DNA chemistry simulation was ended at 2.5 ns in the current work. In the previous work^68^, in which the DNA damage induction algorithm was developed and verified, the chemical stage was continued to 1 *μ*s. As a result, the relatively small number of ^•^OH molecules formed during the chemical stage (by the reaction ${{\rm{e}}}_{{\rm{aq}}}^{-}+{{\rm{H}}}_{2}{{\rm{O}}}_{2}\to {{\rm{OH}}}^{-}+{}^{\bullet }{\rm{O}}{\rm{H}}$, as opposed to during the physicochemical stage) were neglected in the current work. This was predicted to have a negligible effect on the DSB yield, since these ^•^OH molecules are typically formed away from the electron track and thus rarely contribute to clusters of damage (e.g. DSBs).

The DNA damage induction algorithm includes a parameter which may be interpreted as the volume proportion of DNA in the nucleus (DNA volume for short). The DNA damage yields, including the DSB yield, were scaled down according to this parameter. This meant that a fraction of the DSBs within the whole nucleus volume were removed from each cell, chosen at random and irrespective of DSB complexity. Estimates in the literature of the DNA volume in yeast cells vary from 0.3 to 2%^69^. In the current work, the DNA volume was adjusted to achieve realistic DSB yields. Experiments in the literature indicate that the DSB yield increases linearly with dose in the clinically relevant dose range <100 Gy and is generally 20–40/cell/Gy for mammalian haploid cells^7,70–73^. For low-LET radiation and human HNSCC cell lines (*in vitro*) in particular, Saker *et al*.^74^, using colocalization of *γ*H2AX and 53BP1 foci, measured an average of 36 DSBs/cell after 1 Gy with 200 kV x-rays for UTSCC15, FaDuDD and UTSCC14. El-Awady *et al*.^75^ with 220 kV x-rays measured a DSB yield of approximately 20/cell/Gy for SCC4451 using graded-field gel electrophoresis. We adjusted the DNA volume to achieve these yields. The current work includes a sensitivity analysis (see Section: Simulations performed) using DSB yields of approximately 20, 30 and 40/cell/Gy, which were achieved with DNA volumes of 13.3%, 20% and 26.7%, respectively (see Discussion for a possible reason for the high DNA volumes).

### DNA free-end misrejoining and cell death

In our simulation, DNA free-ends from complex DSBs (cDSBs) were able to participate in misrejoining events. To obtain a cDSB yield that was approximately 10% of the DSB yield (and therefore similar to the CL yields in the BIANCA model for x-rays, e.g. 4.0/Gy/cell for a monolayer-shaped fibroblast and 3.1/Gy/cell for a lymphocyte^33^), cDSBs were defined as DSBs with >15 elementary damages (strand breaks, modified bases and modified sugars).

The probability of a misrejoining, *P*_*mr*_, between two incongruent free-ends was modeled as a negative exponential function of the initial distance, *d*, between the two cDSBs that produced them (note that a negative exponential was used in the BIANCA model and the simulated CA yields for low-LET radiation achieved good agreement with experimental data for a monolayer-shaped fibroblast and a lymphocyte^33^):

8 $${P}_{mr}={e}^{-d/{r}_{0}},$$

where *r*_0_ is the characteristic interaction distance, which was an adjustable parameter (e.g.^33^ used values of 0.7 *μ*m for a monolayer-shaped fibroblast and 0.8 *μ*m for a lymphocyte in BIANCA). The parameter *r*_0_ was included in the sensitivity analysis (see Section: Simulations performed).

**Figure 7:**
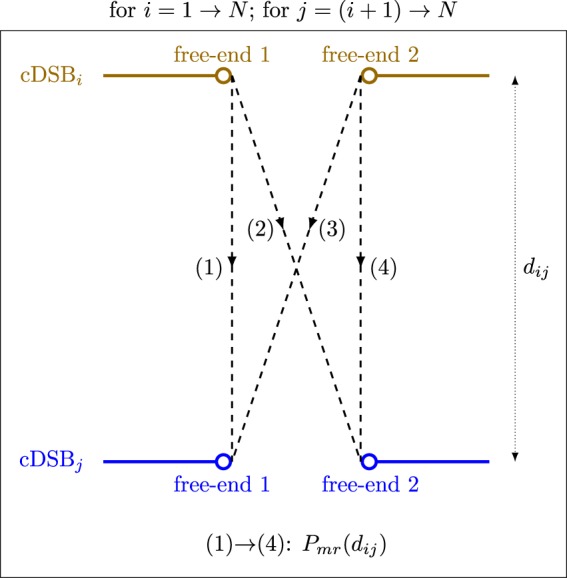
Schematic of the stochastic, proximity-based DNA free-end misrejoining algorithm. A successful misrejoining event removed two free-ends from the pool. *N* is the total number of cDSBs, *d*_*ij*_ is the initial distance between cDSB_*i*_ and cDSB_*j*_, *P*_*mr*_(*d*_*ij*_) is the probability of a misrejoining between a free end of cDSB_*i*_ and a free-end of cDSB_*j*_.

For a nucleus containing *N* cDSBs (cDSB_1_, cDSB_2_, …, cDSB_*N*_), misrejoining was simulated as follows (similar to the method used in the BIANCA model^33^) (Fig. 7): The first free-end of cDSB_1_ had a chance (with probability *P*_*mr*_) to misrejoin with the first free-end of cDSB_2_, then with the second free-end of cDSB_2_. Then the second free-end of cDSB_1_ had a chance to misrejoin with the first free-end of cDSB_2_, then with the second free-end of cDSB_2_. Then the first free-end of cDSB_1_ had a chance to misrejoin with the first free-end of cDSB_3_, then with the second free-end of cDSB_3_, and so on. In general, each free-end of cDSB_*i*_ had a chance to misrejoin with each free-end of cDSB_*i*+1_, cDSB_*i*+2_, …, cDSB_*N*_ (i.e. each combination of free-ends was tried only once). Successful misrejoinings removed free-ends from the “pool” of candidates.

To predict cell death from misrejoinings, the following assumptions were made:

1. No terminal deletions: cDSBs with both ends unrejoined at the end of the simulation were assumed to rejoin faithfully (correctly).
2. No incomplete exchanges: For cDSBs with only one end misrejoined at the end of the simulation, it was assumed the other end misrejoined with the same cDSB as the first, or if that cDSB has no free ends, with the other available end in the misrejoining “chain” (e.g. if cDSB_1_ and cDSB_2_ each have an end misrejoined with an end from cDSB_3_, then the remaining end of cDSB_1_ was assumed to misrejoin with the remaining end of cDSB_2_). However, this was not counted toward the total number of misrejoinings.
3. If two cDSBs misrejoined both of their ends together explicitly during the simulation, the second misrejoining was not counted (it was assumed, from the previous assumption).

With these assumptions in place, each counted misrejoining independently had a 50% chance of resulting in an asymmetric exchange-type CA and thus causing cell death (Supplementary Methods). Accordingly, the probability of cell survival from misrejoining, ${P}_{surv(mr)}$, was related to the total number of counted misrejoinings, *N*_*mr*_, by (similar to Brenner’s model^34^):

9 $${P}_{surv(mr)}={0.5}^{{N}_{mr}}$$

In the sensitivity analysis (see Section: Simulations performed), values other than 0.5 were also considered for the probability that a misrejoining was non-lethal, *P*_*nlmr*_, in which case:

10 $${P}_{surv(mr)}={({P}_{nlmr})}^{{N}_{mr}}$$

### Simulations performed

#### Sensitivity analysis

Doses to the tumor (1224 cells) of up to 1 Gy were simulated with track structure (1 mGy, 3 mGy, 10 mGy, 30 mGy, 60 mGy, 0.1 Gy, 0.3 Gy, 0.5 Gy, 0.7 Gy and 1 Gy). For each dose, DNA damage induction and DNA free-end misrejoining were simulated in all 1224 cells under conditions of full oxia (pO_2_ of 760 mmHg) and anoxia (pO_2_ of 0 mmHg). The number of DSBs per cell vs dose and the number of cDSBs per cell vs dose were fit (in Matlab) to:

11 $${N}_{{\rm{DSB}}}={m}_{{\rm{DSB}}}D$$

12 $${N}_{{\rm{cDSB}}}={m}_{{\rm{cDSB}}}D$$

The number of misrejoinings per cell vs dose was fit to:

13 $${N}_{mr}={\alpha }_{mr}D+{\beta }_{mr}{D}^{2}$$

If *α*_*mr*_ < 0, the fit was reperformed to:

14 $${N}_{mr}={\beta }_{mr}{D}^{2}$$

The mean probability of cell survival from misrejoining was fit to:

15 $${P}_{surv(mr)}={e}^{-{\alpha }_{{\rm{killing}}(mr)}D-{\beta }_{{\rm{killing}}(mr)}{D}^{2}}$$

If ${\alpha }_{{\rm{killing}}(mr)} < 0$, the fit was reperformed to:

16 $${P}_{surv(mr)}={e}^{-{\beta }_{{\rm{killing}}(mr)}{D}^{2}}$$

The oxygen enhancement ratios (OERs) for DSB and cDSB induction were obtained by:

17 $${{\rm{OER}}}_{{\rm{DSB}}}=\frac{{m}_{{\rm{DSB}}}({{\rm{pO}}}_{2}\,{\rm{of}}\,760\,{\rm{mmHg}})}{{m}_{{\rm{DSB}}}({{\rm{pO}}}_{2}\,{\rm{of}}\,0\,{\rm{mmHg}})}$$

18 $${{\rm{OER}}}_{{\rm{cDSB}}}=\frac{{m}_{{\rm{cDSB}}}({{\rm{pO}}}_{2}\,{\rm{of}}\,760\,{\rm{mmHg}})}{{m}_{{\rm{cDSB}}}({{\rm{pO}}}_{2}\,{\rm{of}}\,0\,{\rm{mmHg}})}$$

The surviving fraction from misrejoining after a dose of 2 Gy, SF_2(*mr*)_, was obtained by extrapolation using the fit for ${P}_{surv(mr)}$. The OER for cell killing by misrejoining was measured as:

19 $${{\rm{OER}}}_{{\rm{killing}}(mr)}=\frac{{D}_{10}({{\rm{pO}}}_{2}\,{\rm{of}}\,0\,{\rm{mmHg}})}{{D}_{10}({{\rm{pO}}}_{2}\,{\rm{of}}\,760\,{\rm{mmHg}})}$$

where the dose to achieve 10% survival, *D*_10_, was obtained by extrapolation using the fit for ${P}_{surv(mr)}$.

A sensitivity analysis was performed for the model parameters DSB yield ∈ {20.1, 30.1, 40.2}/cell/Gy, *r*_0_ ∈ {0.5, 0.7, 0.9} *μ*m and *P*_*nlmr*_ ∈ {0.25, 0.5, 0.75}, using the endpoints OER_DSB_, OER_cDSB_, *α*_*mr*_, *β*_*mr*_, ${\alpha }_{{\rm{killing}}(mr)}$, ${\beta }_{{\rm{killing}}(mr)}$, SF_2(*mr*)_ and ${{\rm{OER}}}_{{\rm{killing}}(mr)}$.

Simulations were performed on the Phoenix supercomputer (University of Adelaide, Adelaide, Australia). To irradiate the tumor (1224 cells) with tracks to a uniform dose of 1 Gy required 637 runs of 5 × 10^7^ x-rays each. Each run required 5 cores sharing 30 GB of memory for 1.25 hours (4000 core hours total). The amount of resources required to run the the DNA damage induction algorithm was non-linear with dose. For a dose of 1 Gy, the DNA damage algorithm required 1 core with 35 GB for 8 hours, followed by 5 cores sharing 110 GB for 32 hours, once for full oxia and once for anoxia (328 core hours total). For a dose of 0.7 Gy, it used 1 core with 25 GB for 3 hours, followed by 5 cores sharing 50 GB for 13 hours, once for full oxia and once for anoxia (29 core hours total). The misrejoining algorithm required almost no resources.

### Isolation of direct-type effects

To investigate the contributions from direct-type and indirect effects, DNA damage induction and DNA free-end misrejoining were repeated for each dose without including the indirect effect of DNA damage induction (i.e. ^•^OH interactions with DNA). A DSB yield of 30.1/Gy/cell, *r*_0_ of 0.7 *μ*m and P_*nlmr*_ of 0.5 were used. Without the indirect (^•^OH) events, the DNA damage algorithm required approximately half as many core hours and GB of memory.

### 1 Gy dose delivered to HNSCC tumor of volume 1 mm^3^

The voxelized tumor (the cubic volume of side length 0.2 mm containing 1224 cells) was replicated 125 times to form a cubic volume of side length 1 mm^3^ containing $1224\times \mathrm{125=153000}$ cells. A previously developed model for cellular HNSCC tumor growth with angiogenesis^55,76^ was used to generate a connected network of blood vessels through the 1 mm^3^ tumor and then assign each cell with a pO_2_ according to its proximity to blood vessels (chronic hypoxia).

The relevant model parameters were the relative vascular volume (*RVV*), the blood oxygenation (*p*_0_) and the distance from a vessel to the onset of necrosis (necrosis distance, *ND*). While HNSCC can have *RVV* from 2–10%, *p*_0_ from 20–100 mmHg and *ND* from 80–300 *μ*m^77^, a typical HNSCC is very hypoxic, requiring these parameters to take values near their lower limits^76^. In order to achieve a typical HNSCC oxygenation (see Results; Section: 1 Gy dose delivered to HNSCC tumor of volume 1 mm^3^), an *RVV* of 2.1%, *p*_0_ of 30 mmHg and *ND* of 130 *μ*m were chosen.

The same physical and chemical tracks through the original 1224 cells from 1 Gy dose were used for all 125 “copies” of the (0.2 mm)^3^ tumor. The positions that were occupied by viable cells (as opposed to necrotic cells or blood vessel units) and their pO_2_ values differed between copies. Stochastic DNA damage induction and subsequent DNA free-end misrejoining were simulated for each viable (non-necrotic) cell in each copy. While the spatial distributions of direct and indirect events in nuclei were the same between copies, the DNA targets were moved each time, affecting which direct and indirect events hit the DNA. Furthermore, the same event on DNA may have produced a DNA radical on the sugar moiety one time and on a base the next. Most importantly, a higher cellular pO_2_ made it more likely for a DNA radical to be converted to a strand break. For all of these reasons, the spatial distribution of DSBs in each nucleus was different between copies.

For the 1 mm^3^ tumor of HNSCC, bivariate frequency distributions were generated of (i) the cellular pO_2_ and the number of DSBs in the nucleus, (ii) the cellular pO_2_ and the number of cDSBs in the nucleus, (iii) the cellular pO_2_ and the number of misrejoinings in the nucleus and (iv) the cellular pO_2_ and the cell survival probability from misrejoining.

The DNA damage algorithm required 1 core with 35 GB for 8 hours, followed by 5 cores sharing 110 GB for 32 hours for each of the 125 copies (20,008 core hours in total).

## Results

### Sensitivity analysis

**Figure 8:**
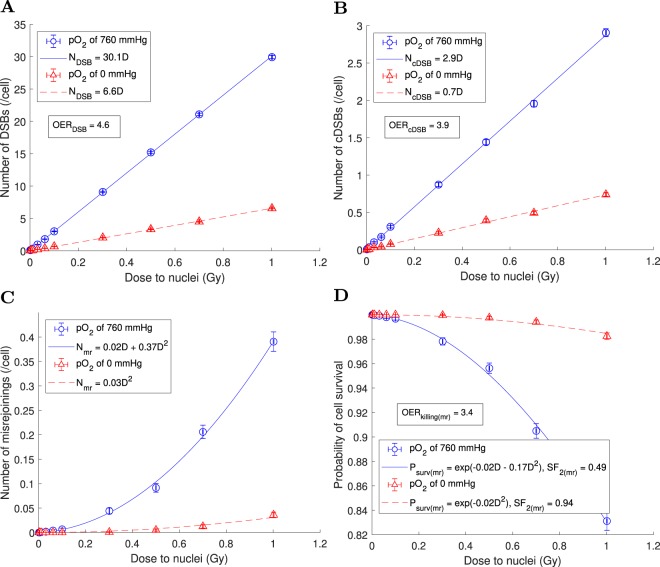
Variation with dose of the mean values of cell endpoints, obtained using a DSB yield of 30.1/Gy/cell, *r*_0_ of 0.7 *μ*m and ${P}_{nlrm}$ of 0.5. (**A**) Number of DSBs per cell vs dose. (**B**) Number of cDSBs per cell vs dose. (**C**) Number of misrejoinings per cell vs dose. (**D**) Cell survival probability from misrejoining vs dose. The mean values for *n* = 1224 cells were plotted with error bars equal to the SEM.

Variation with dose of the number of DSBs per cell, the number of cDSBs per cell, the number of misrejoinings per cell and the mean cell survival probability from misrejoining, under full oxia and anoxia, are shown in Fig. 8. These results were obtained using a DSB yield of 30.1/Gy/cell, *r*_0_ of 0.7 *μ*m and ${P}_{nlrm}$ of 0.5. The number of misrejoinings per cell and the cell survival probability from misrejoining were mostly quadratic with dose under full oxia (${\alpha }_{mr}=0.02$ Gy^−1^ and ${\beta }_{mr}=0.37$ Gy^−2^; ${\alpha }_{{\rm{killing}}(mr)}=0.02$ Gy^−1^ and ${\beta }_{{\rm{killing}}(mr)}=0.17$ Gy^−2^) and purely quadratic under anoxia (${\beta }_{mr}=0.03$ Gy^−2^; ${\beta }_{{\rm{killing}}(mr)}=0.02$ Gy^−2^).

The results of a sensitivity analysis for the parameters DSB yield, *r*_0_ and ${P}_{nlrm}$ are presented in Tables 3–5, respectively. The amount of misrejoining and hence cell killing increased with increasing DSB yield and *r*_0_ while the amount of cell killing decreased with increasing ${P}_{nlrm}$, as expected. Note that misrejoining and cell killing consistently had small linear components (i.e. the ${\alpha }_{mr}$ and ${\alpha }_{{\rm{killing}}(mr)}$ values). Also consistently observed were ${{\rm{OER}}}_{{\rm{cDSB}}} < {{\rm{OER}}}_{{\rm{DSB}}}$ and ${{\rm{OER}}}_{{\rm{killing}}(mr)} < {{\rm{OER}}}_{{\rm{DSB}}}$. For the remainder of the results, a DSB yield of 30.1/Gy/cell, *r*_0_ of 0.7 *μ*m and ${P}_{nlrm}$ of 0.5 were used.

**Table 3:** Effects of varying the DSB yield.

DSB/cDSB yields^a^ (/Gy/cell) under full oxia	OER_DSB_	OER_cDSB_	*α*_*mr*_ (Gy^−1^)/*β*_*mr*_ (Gy^−2^) under full oxia (anoxia)^b,c^	*α*_killing(*mr*)_ (Gy^−1^)/*β*_killing(*mr*)_ (Gy^−2^) under full oxia (anoxia)^d,e^	SF_2(*mr*)_ under full oxia (anoxia)^f,e^	OER_killing(*mr*)_^g,e^
20.1/1.9	4.6	3.9	0^h^/0.19 (0^h^/0.01)	0^i^/0.09 (0^i^/0.01)	0.70 (0.97)	3.6
30.1/2.9	4.6	3.9	0.02/0.37 (0^h^/0.03)	0.02/0.17 (0^i^/0.02)	0.49 (0.94)	3.4
40.2/3.9	4.6	4.0	0.07/0.56 (0.00/0.04)	0.04/0.25 (0.00/0.02)	0.34 (0.92)	3.6

^a^Using the slopes of the fits $${N}_{DSB}={m}_{DSB}D$$ and $${N}_{cDSB}={m}_{cDSB}D$$. ^b^Fit to $${N}_{mr}={\alpha }_{mr}D+{\beta }_{mr}{D}^{2}$$. ^c^Using *r*_0_ = 0.7 *μ*m. ^d^Fit to $${P}_{surv(mr)}={e}^{-{\alpha }_{{\rm{killing}}(mr)}D-{\beta }_{{\rm{killing}}(mr)}{D}^{2}}$$. ^e^Using *r*_0_ = 0.7 *μ*m and $${P}_{nlmr}=0.5$$. ^f^Using the fit to $${P}_{surv(mr)}$$ and extrapolating to 2 Gy. ^g^Using $${{\rm{OER}}}_{{\rm{killing}}(mr)}={D}_{10}({{\rm{pO}}}_{2}\,{\rm{of}}\,0\,{\rm{mmHg}})/$$$${D}_{10}({{\rm{pO}}}_{2}\,{\rm{of}}\,760\,{\rm{mmHg}})$$, where *D*_10_ was obtained by extrapolating the fit to $${P}_{surv(mr)}$$. ^h^The fit gave $$-0.01\lesssim {\alpha }_{mr} < 0$$, so the fit was reperformed to $${N}_{mr}={\beta }_{mr}{D}^{2}$$. ^i^The fit gave $$-0.01 < {\alpha }_{{\rm{killing}}(mr)} < 0$$, so the fit was reperformed to $${P}_{surv(mr)}={e}^{-{\beta }_{{\rm{killing}}(mr)}{D}^{2}}$$.

**Table 4:** Effects of varying the characteristic interaction distance, *r*_0_ (from Eq. 8). A DSB yield of 30.1/Gy/cell (cDSB yield of 2.9/Gy/cell) under full oxia was used.

*r*_0_ (*μ*m)	*α*_*mr*_ (Gy^−1^)/*β*_*mr*_ (Gy^−2^) under full oxia (anoxia)^a^	*α*_killing(*mr*)_ (Gy^−1^)/*β*_killing(*mr*)_ (Gy^−2^) under full oxia (anoxia)^b,c^	SF_2(*mr*)_ under full oxia (anoxia)^d,c^	OER_killing(*mr*)_^e,c^
0.5	0.01/0.17 (0^f^/0.01)	0.01/0.08 (0^g^/0.01)	0.71 (0.97)	3.5
0.7	0.02/0.37 (0^f^/0.03)	0.02/0.17 (0^g^/0.02)	0.49 (0.94)	3.4
0.9	0.04/0.54 (0^f^/0.04)	0.03/0.24 (0^g^/0.02)	0.36 (0.92)	3.4

^a^Fit to ${N}_{mr}={\alpha }_{mr}D+{\beta }_{mr}{D}^{2}$. ^b^Fit to ${P}_{surv(mr)}={e}^{-{\alpha }_{{\rm{killing}}(mr)}D-{\beta }_{{\rm{killing}}(mr)}{D}^{2}}$. ^c^Using ${P}_{nlmr}=0.5$. ^d^Using the fit to ${P}_{surv(mr)}$ and extrapolating to 2 Gy. ^e^Using ${{\rm{OER}}}_{{\rm{killing}}(mr)}={D}_{10}({{\rm{pO}}}_{2}\,{\rm{of}}\,0\,{\rm{mmHg}})/{D}_{10}({{\rm{pO}}}_{2}\,{\rm{of}}\,760\,{\rm{mmHg}})$, where *D*_10_ was obtained by extrapolating the fit to ${P}_{surv(mr)}$. ^f^The fit gave $-0.01\lesssim {\alpha }_{mr}\mathrm{ < 0}$, so the fit was reperformed to ${N}_{mr}={\beta }_{mr}{D}^{2}$. ^g^The fit gave $-0.01 < {\alpha }_{{\rm{killing}}(mr)} < 0$, so the fit was reperformed to ${P}_{surv(mr)}={e}^{-{\beta }_{{\rm{killing}}(mr)}{D}^{2}}$.

**Table 5:** Effects of varying the probability that a misrejoining was non-lethal, *P*_*nlmr*_ (from Eq. 10**)**.

*P*_*nlmr*_	*α*_killing(*mr*)_ (Gy^−1^)/*β*_killing(*mr*)_ (Gy^−2^) under full oxia (anoxia)^a^	SF_2(*mr*)_ under full oxia (anoxia)^b^	OER_killing(*mr*)_^c^
0.25	0.03/0.25 (0^d^/0.02)	0.35 (0.91)	3.3
0.5	0.02/0.17 (0^d^/0.02)	0.49 (0.94)	3.4
0.75	0.01/0.09 (0^d^/0.01)	0.69 (0.97)	3.4

A DSB yield of 30.1/Gy/cell (cDSB yield of 2.9/Gy/cell) under full oxia and *r*_0_ of 0.7 *μ*m were used.

^a^Fit to ${P}_{surv(mr)}={e}^{-{\alpha }_{{\rm{killing}}(mr)}D-{\beta }_{{\rm{killing}}(mr)}{D}^{2}}$. ^b^Using the fit to ${P}_{surv(mr)}$ and extrapolating to 2 Gy. ^c^Using ${{\rm{OER}}}_{{\rm{killing}}(mr)}={D}_{10}({{\rm{pO}}}_{2}\,{\rm{of}}\,0\,{\rm{mmHg}})/{D}_{10}({{\rm{pO}}}_{2}\,{\rm{of}}\,760\,{\rm{mmHg}})$, where *D*_10_ was obtained by extrapolating the fit to ${P}_{surv(mr)}$. ^d^The fit gave $-0.01 < {\alpha }_{{\rm{killing}}(mr)} < 0$, so the fit was reperformed to ${P}_{surv(mr)}={e}^{-{\beta }_{{\rm{killing}}(mr)}{D}^{2}}$.

**Figure 9:**
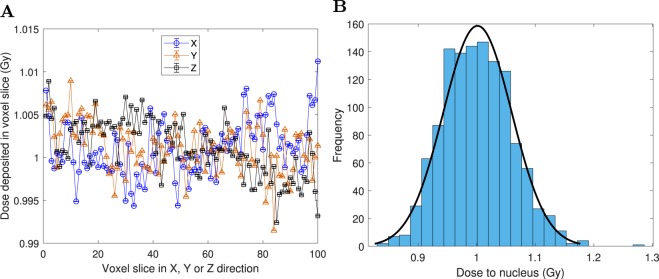
1 Gy dose delivered to the tumor (1224 cells) with track structure. (**A**) Spatial dose profiles, using voxel slices in the X, Y and Z directions. The mean dose for *n* = 10^4^ voxels in a voxel slice was plotted with vertical error bars equal to the SEM (which were smaller than the markers). (**B**) Frequency distribution of dose to nuclei (*n* = 1224), with a normal distribution fit overlaid.

For the 1 Gy irradiation, the dose to the tumor was uniform to within 1% (Fig. 9A) and the dose to nuclei (*n* = 1224) was approximately normally distributed with a mean and standard deviation of 1.00 ± 0.06 Gy (Fig. 9B).

**Figure 10:**
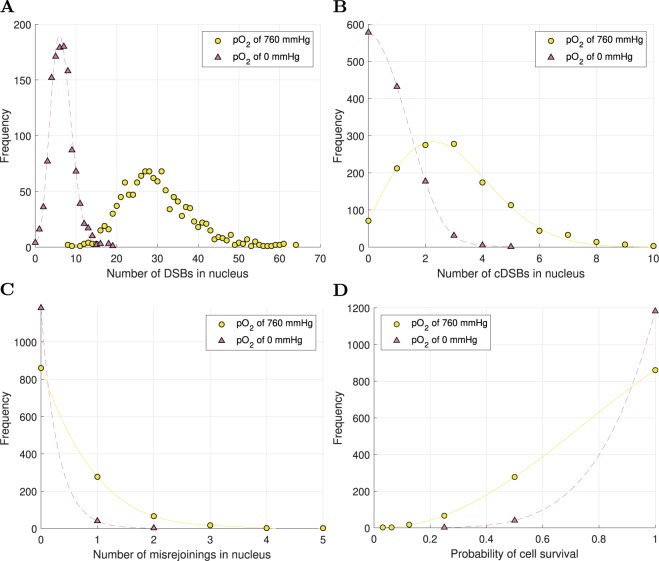
Frequency distributions of cell endpoints in *n* = 1224 cells for 1 Gy dose, under full oxia and anoxia. (**A**) Number of DSBs in nuclei. (**B**) Number of cDSBs in the nuclei. (**C**) Number of misrejoinings in the nuclei. (**D**) Cell survival probability from misrejoining.

Frequency distributions of the number of DSBs in nuclei, the number of cDSBs in nuclei, the number of misrejoinings in nuclei and the cell survival probability from misrejoining, under full oxia and anoxia, are shown in Fig. 10 for the 1 Gy dose.

**Figure 11:**
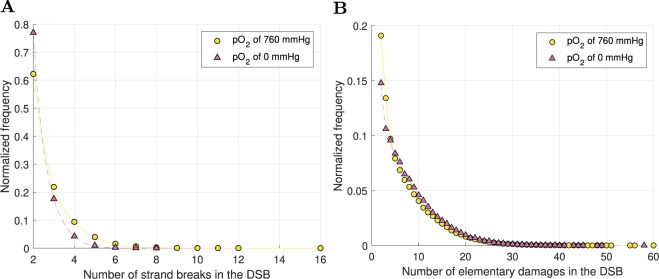
Frequency distributions of DSB properties for 1 Gy dose, under full oxia and anoxia. (**A**) Number of strand breaks in DSBs. (**B**) Number of elementary damages (strand breaks, modified bases and modified sugars) in DSBs. The total number of DSBs in 1224 cells was $n\mathrm{=187518}$ under full oxia and $n\mathrm{=40886}$ under anoxia. The frequency distributions were normalized for ease of comparison between full oxia and anoxia.

Figure 11 provides information about the complexity of the DSBs induced under full oxia and anoxia. While there were fewer DSBs under anoxia, the DSBs were on average more complex in terms of the number of elementary damages (strand breaks, modified bases and modified sugars). Given how cDSBs were defined (DSBs with >15 elementary damages), this is consistent with ${{\rm{OER}}}_{{\rm{cDSB}}} < {{\rm{OER}}}_{{\rm{DSB}}}$. Almost all DSBs had unit multiplicity (98.3% of DSBs under full oxia and 99.6% of DSBs under anoxia).

### Isolation of direct-type effects

If DNA damage from the indirect effect was not simulated, the DSB yield was reduced to approximately a third of its original value and the cDSB yield was reduced to a tenth of its original value (Table 6).

**Table 6:** The impact of neglecting the indirect effect.

	DSB/cDSB yields (/Gy/cell) under full oxia	OER_DSB_	OER_cDSB_
D^a^ + I^b^	30.1/2.9	4.6	3.9
D^a^ only	12.3/0.3	2.6	2.2

^a^Direct-type effects. ^b^Indirect effect.

### 1 Gy dose delivered to HNSCC tumor of volume 1 mm^3^

**Figure 12:**
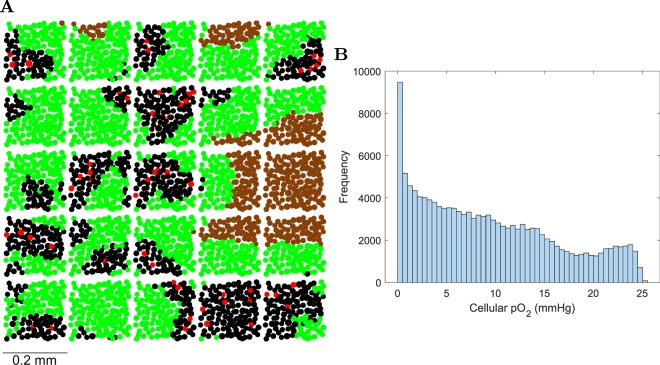
1 mm^3^ tumor of HNSCC. (**A**) Section (thickness 20 *μ*m) showing blood vessel units (red), normoxic cells (pO_2_ > 10 mmHg) (black) located near the blood vessels, hypoxic cells (pO_2_ ≤ 10 mmHg) (green) located further from the blood vessels and necrotic cells (brown) located further than *ND* = 130 *μ*m from a blood vessel. It can be seen how the 1 mm^3^ tumor was constructed by replicating the (0.2 mm)^3^ tumor. (**B**) Frequency distribution of pO_2_ for viable (non-necrotic) cells ($n=135306$) in the 1 mm^3^ tumor of HNSCC.

A 1 mm^3^ tumor was generated (Fig. 12A) with a cellular pO_2_ distribution (Fig. 12B) characteristic of HNSCC ^76^: the mean cellular pO_2_ was 9.4 mmHg, the median cellular pO_2_ was 8.2 mmHg, the HP_10_ (proportion of viable cells with p O_2_ < 10 mmHg) was 58%, the HP_5_ was 34%, the HP_2.5_ was 20%, the HP_1_ was 11% and the necrotic volume was 9.4%.

**Figure 13:**
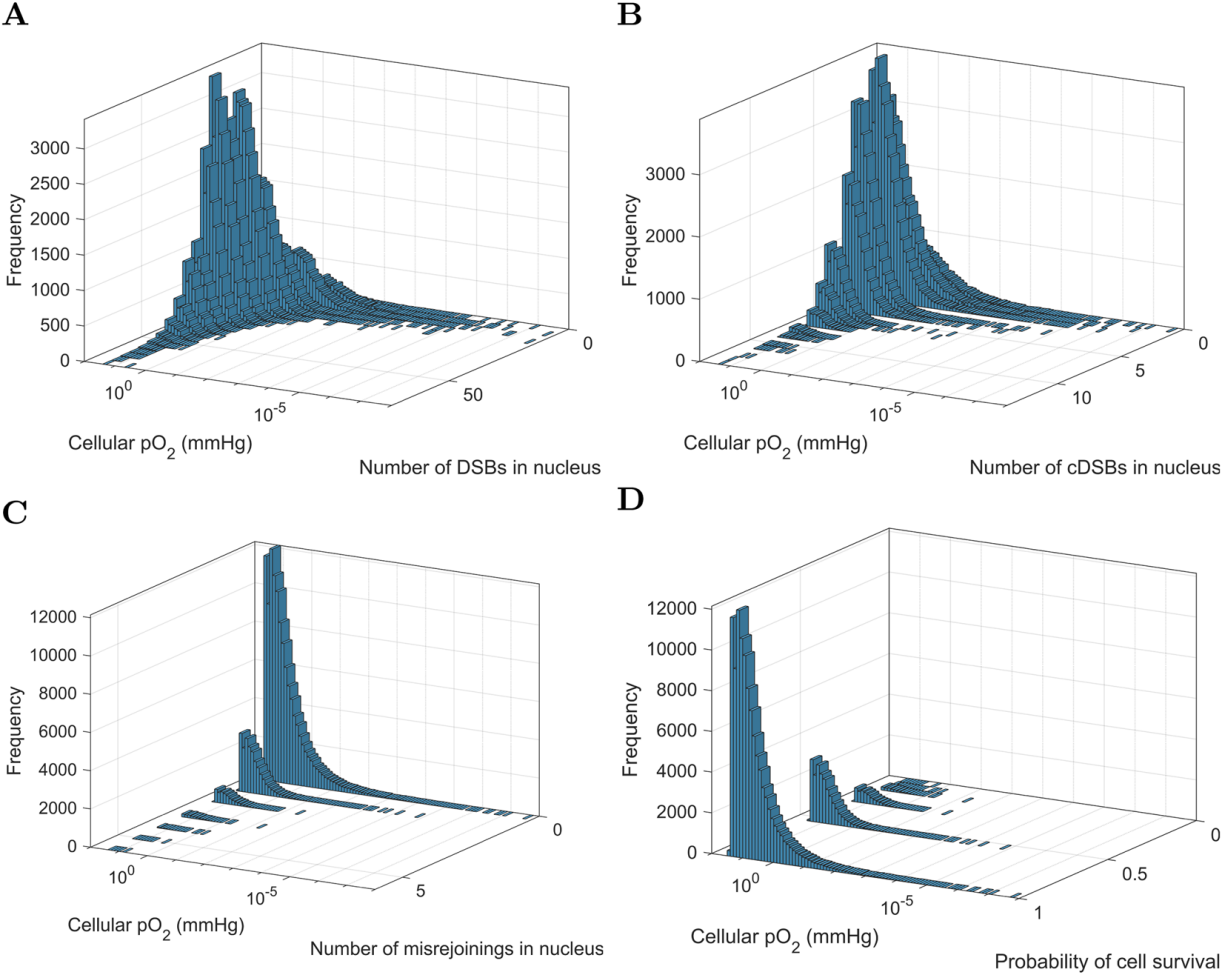
Bivariate frequency distributions of cellular pO_2_ and cell endpoints in $n=135306$ viable cells of the 1 mm^3^ HNSCC tumor for 1 Gy dose. (**A**) Cellular pO_2_ and the number of DSBs in the nucleus. (**B**) Cellular pO_2_ and the number of cDSBs in the nucleus. (**C**) Cellular pO_2_ and the number of misrejoinings in the nucleus. (**D**) Cellular pO_2_ and the cell survival probability from misrejoining.

Figure 13 shows, for 1 Gy dose, bivariate frequency distributions of the cellular pO_2_ and the number of DSBs in the nucleus, the cellular pO_2_ and the number of cDSBs in the nucleus, the cellular pO_2_ and the number of misrejoinings in the nucleus and the cellular pO_2_ and the cell survival probability from misrejoining.

## Discussion

DNA free-end misrejoining and its contribution to cell killing for MV x-rays was investigated. The linear components of misrejoining (${\alpha }_{mr}$) and cell killing from misrejoining (${\alpha }_{{\rm{killing}}(mr)}$) were consistently small in the sensitivity analysis. For example, for a DSB yield of 30.1/Gy/cell, *r*_0_ of 0.7 *μ*m and ${P}_{nlmr}$ of 0.5, ${\alpha }_{{\rm{killing}}(mr)}$ was 0.02 Gy^−1^ under full oxia (and zero under anoxia). For comparison, a typical ${\alpha }_{{\rm{killing}}}$ for HNSCC is 0.3 Gy^−1^ ^41,78–82^. This indicated that (i) misrejoinings involving DSBs from the same primary were a very rare occurrence, which is in agreement with deterministic modeling by Carlson *et al*.^26^, and (ii) other mechanisms may be chiefly responsible for the linear components of CA production and cell killing by low-LET radiation for HNSCC. Unrejoined DNA free-ends (terminal deletions and incomplete exchanges) are likely candidates. It has been estimated that approximately 5% of DSBs end up as either terminal deletions or incomplete exchanges for low-LET radiation^8^.

On the other hand, the quadratic components of misrejoining (${\beta }_{mr}$) and cell killing from misrejoining (${\beta }_{{\rm{killing}}(mr)}$) were consistently large in the sensitivity analysis. For the aforementioned combination of {DSB yield, *r*_0_, ${P}_{nlmr}\}$, ${\beta }_{{\rm{killing}}(mr)}$ was 0.17 Gy^−2^ under full oxia (0.02 Gy^−2^ under anoxia), whereas a typical value of ${\beta }_{{\rm{killing}}}$ for HNSCC is 0.03 Gy^−2^ ^41,78–83^. This indicates that too much misrejoining was simulated (which makes the small linear component of cell killing by misrejoining look even smaller). Consistent with this notion, the SF_2(*mr*)_ under full oxia was already 49% without simulating terminal deletions and incomplete exchanges as sources of cell killing.

The amount of misrejoining could be reduced by decreasing the model parameter *r*_0_ below 0.5 *μ*m. However, this may not be optimal, since the BIANCA model obtained good results for the CA yields in a monolayer-shaped fibroblast using an *r*_0_ of 0.7 *μ*m and in a lymphocyte using an *r*_0_ of 0.8 *μ*m^33^. Alternatively, misrejoining could be reduced by increasing the size of the nuclei. This may be well advised given that large DNA volumes from 13.3 to 26.7% were required to obtain DSB yields from 20 to 40/Gy/cell (for reference, estimates of the DNA volume in yeast cells vary from 0.3 to 2%^69^). Increasing the nucleus volume would require a proportional decrease in the DNA volume to obtain the same DSB yield. However, a large DNA volume was also encountered in the computational model by Henthorn *et al*.^28^. Their model, like the current one, used a spatial clustering approach to predict DNA damage. They required the sensitive fraction of the nucleus to be 15% to achieve a realistic DSB yield.

It is also possible that some misrejoinings were counted that should not have been. If a group of $N\ge 3$ breaks involved *N* explicitly simulated misrejoinings (the maximum number), a value of ${N}_{mr}=N$ was mistakenly used in Eq. 9 (or Eq. 10) for the cell survival probability; the true probability in this case is obtained by using a value of ${N}_{mr}=N-1$ (Supplementary Methods). However, for the largest dose simulated (1 Gy), only 1.7% of cells (22/1224 cells) under full oxia (and no cells under axoia) contained 3 or more misrejoinings (Fig. 10C). Therefore, these erroneous cases could not have contributed appreciably to the current results. This oversight will be rectified in future iterations of the model. It is expected to be important for simulating high dose fractions and high-LET proton and carbon beams, which are planned for future work.

A limitation of the model was the assumption of no incomplete exchanges and no terminal deletions. There would be greater cell killing if these mechanisms were accounted for. Another limitation was that a negative exponential function was used to model the probability of misrejoining (Eq. 8). This was an approximation of complex DSB repair processes (e.g. c-NHEJ), which involve cascades of a large number of DNA repair proteins^13^. Additionally, there can be DNA damage from non-targeted effects^24^, which were not considered. The model also neglected cell death by apoptosis, when apoptosis is not a consequence of lethal CA-driven mitotic catastrophe^1,21–24^.

This work presented a new stochastic model of radiation-induced CA production and cell death that addressed a gap in the literature. Rather than focussing on simulating a complex DNA target model (e.g. Friedland and Kundrát’s model^29,30^, the BIANCA model^31–33^), or the motion of DNA free-ends (Friedland and Kundrát, the model by Henthorn *et al*. ^28^, Brenner’s model^34^), or mechanistic modeling of NHEJ (Henthorn *et al*., Friedland and Kundrát), the current model uniquely simulated Monte Carlo track structure through a multicellular tumor volume, and converted the physical and chemical tracks in nuclei into DNA damage in a pO_2_-dependent fashion. These are important developments toward a spatio-temporal, multicellular, track structure-based radiotherapy model^55,76^.

## Conclusion

The current work achieved the stochastic simulation of (i) x-ray irradiation of a multicellular tumor, (ii) pO_2_-dependent DNA damage induction from the tracks, (iii) proximity-based DNA free-end misrejoining and (iv) cell death from misrejoining. The model was used to investigate the contribution of misrejoining to cell killing in isolation for MV x-rays. A sensitivity analysis was conducted for the model parameters DSB yield, characteristic interaction distance and probability that a misrejoining was non-lethal. As an application, DNA damage and misrejoining were simulated in 135306 viable cells in a 1 mm^3^ hypoxic HNSCC tumor for a uniform dose of 1 Gy. The near absence of linear components of misrejoining and cell killing from misrejoining suggests that terminal deletions and incomplete exchanges may be important mechanisms for the linear components of CA production and cell killing by low-LET radiation for HNSCC, under the assumptions of the current model.

## Supplementary information

Supplementary Information
