# Supplementary material for: Stochastic multicellular modeling of x-ray irradiation, DNA damage induction, DNA free-end misrejoining and cell death
Source: Sci Rep. 2019 Dec 11;9:18888. doi: 10.1038/s41598-019-54941-1 (PMC6906404; doi:10.1038/s41598-019-54941-1)
Supplement: Supplementary file 1 — Supplementary Information [file 41598_2019_54941_MOESM1_ESM.pdf]

Supplementary Methods

J.C. Forster, M.J.J. Douglass, W.M. Phillips, E. Bezak

Prediction of cell death from DNA free-end misrejoining

This appendix provides the justification for

$$P_{surv(mr)} = 0.5^{N_{mr}}$$

The assumptions that were made in this model are:

1. No terminal deletions: cDSBs with both ends unrejoined were rejoined faithfully/correctly.

2. No incomplete exchanges: For cDSBs with only one end misrejoined, assume the other end misrejoined with the same cDSB as the first, or if that cDSB has no free ends, with the other available end in the misrejoining "chain" (e.g. if $cDSB_1$ and $cDSB_2$ each have an end misrejoined with an end from $cDSB_3$, then the remaining end of $cDSB_1$ was assumed to misrejoin with the remaining end of $cDSB_2$).

3. If two cDSBs misrejoined both of their ends together during the original misrejoining simulation, the second misrejoining was not counted, as this was assumed (from the previous assumption).

Let the number of breaks (DSBs, or cDSBs in the current work) in the nucleus be $N \in [2, \infty)$. The number of misrejoinings involving these breaks is $N_{mr} \in [0, N]$. It will be demonstrated that this equation for $P_{surv(mr)}$ is true (given the assumptions above) for $N = 2$ and all $N_{mr} \in \{0, 1, 2\}$ and for $N \geq 3$ provided $N_{mr} \neq N$. For $N \geq 3$ and $N_{mr} = N$, one of the misrejoinings was counted that should not have been, i.e., $P_{surv(mr)}$ would have been correct if a value of $N_{mr} \to N_{mr} - 1$ had been used in the formula instead. Thus, the cell killing was overestimated in these cases (though they did not contribute appreciably in the current work - see Discussion). Lastly, it will be shown that the equation for $P_{surv(mr)}$ extends to a nucleus containing multiple independent groups of breaks with misrejoinings.

$N = 2$

2 breaks on 2 chromosomes (1 break on each chromosome)

If there were no misrejoinings ($N_{mr} = 0$), both breaks were rejoined faithfully (assumption 1) and $P_{surv(mr)} = 1$, which the formula gives for $N_{mr} = 0$.

If there was 1 misrejoining ($N_{mr} = 1$), the options are:

- 1 dicentric (with no sticky ends) and 2 acentric fragments (each with 1 sticky end) (1 way)
- 1 acentric fragment (with no sticky ends) and 2 centric fragments (each with 1 sticky end) (1 way)
- 1 symmetric translocation (with no sticky ends), 1 centric fragment (with 1 sticky end) and 1 acentric fragment (with 1 sticky end) (2 ways)

A second misrejoining was then assumed (assumption 2), giving either:

- 1 dicentric (no sticky ends) and 1 acentric fragment (no sticky ends) (2 ways) $\rightarrow$ lethal
- 2 symmetric translocations (no sticky ends) (2 ways) $\rightarrow$ viable

Thus there were 2 equally likely outcomes (2 ways each): 1 lethal and 1 viable. Therefore $P_{surv(mr)} = 0.5$, which the formula gives for $N_{mr} = 1$.

If there were 2 misrejoinings simulated explicitly, the situation was described above and $P_{surv(mr)} = 0.5$. However, because the 2 simulated misrejoings were between the same 2 breaks, only the first misrejoining was counted toward N_{mr} (assumption 3), i.e., $N_{mr} = 1$ was used in the formula and thus the correct answer of $P_{surv(mr)} = 0.5$ was obtained.

1 break on each arm of a chromosome

If $N_{mr} = 0$, same as in the previous example.

If there was one misrejoining ($N_{mr} = 1$), the options are:

- 1 centric ring and 2 acentric fragments (each with 1 sticky end) (1 way)
- 1 acentric fragment (no sticky ends) and 1 centric fragment (2 sticky ends) (1 way)
- half of a pericentric inversion, giving 1 centric fragment (1 sticky end) and 1 acentric fragment (1 sticky end) (2 ways)

A second misrejoining was then assumed (assumption 2), giving either:

- 1 centric ring and 1 acentric fragment (no sticky ends) (2 ways) $\rightarrow$ lethal
- pericentric inversion (2 ways) $\rightarrow$ viable

Lethal and viable outcomes were equally likely (2 ways each), so $P_{surv(mr)} = 0.5$, which the formula gives for $N_{mr} = 1$.

As in the previous example, if the 2 misrejoinings were simulated explicitly, the second misrejoining was not counted toward N_{mr} (assumption 3), so $N_{mr} = 1$ and the formula correctly gives $P_{surv(mr)} = 0.5$.

2 breaks on the same arm of a chromosome

If $N_{mr} = 0$, same as above.

If there was one misrejoining ($N_{mr} = 1$), the options are:

- interstitial deletion, giving 1 acentric fragment (2 sticky ends) and 1 centric fragment (no sticky ends) (1 way)
- 1 acentric ring, 1 centric fragment (1 sticky end) and 1 acentric fragment (1 sticky end) (1 way)
- half of a paracentric inversion, giving 1 centric fragment (1 sticky end) and 1 acentric fragment (1 sticky end) (2 ways)

A second misrejoining was then assumed (assumption 3), giving either:

- interstitial deletion, with 1 acentric ring and 1 centric fragment (no sticky ends) (2 ways) $\rightarrow$ lethal
- paracentric inversion (2 ways) $\rightarrow$ viable

Lethal and viable outcomes were equally likely (2 ways each), so $P_{surv(mr)} = 0.5$, which the formula gives for $N_{mr} = 1$.

As above, if the 2 misrejoinings were simulated explicitly, the second misrejoining was not counted toward N_{mr} (assumption 3), so $N_{mr} = 1$ and the formula correctly gives $P_{surv(mr)} = 0.5$.

$N \geq 3$

3 breaks on 3 chromosomes (1 break on each chromosome)

If there were no misrejoinings ($N_{mr} = 0$), all 3 breaks were rejoined faithfully (assumption 1) and $P_{surv(mr)} = 1$, which the formula gives for $N_{mr} = 0$.

If there was 1 misrejoining ($N_{mr} = 1$), the options are:

- 1 dicentric (no sticky ends), 1 centric fragment (1 sticky end) and 3 acentric framgents (each with 1 sticky end) (3 ways)
- 1 acentric fragment (no sticky ends), 3 centric fragments (each with 1 sticky end) and 1 acentric fragment (1 sticky end) (3 ways)
- 1 symmetric translocation (no sticky ends), 2 centric fragments (each with 1 sticky end) and 2 acentric framgents (each with 1 sticky end) (6 ways)

A second misrejoining was then assumed between the same two breaks as the first (assumption 2), giving options:

- 1 dicentric (no sticky ends), 1 acentric fragmemt (no sticky ends), 1 centric fragment (1 sticky end) and 1 acentric fragment (1 sticky end) (6 ways)
- 2 symmetric translocations (no sticky ends), 1 acentric fragment (1 sticky end) and 1 centric fragment (1 sticky end) (6 ways)

The break that was not involved in any misrejoinings was rejoined faithfully (assumption 1), giving options:

- 1 dicentric (no sticky ends), 1 acentric fragmemt (no sticky ends) and 1 repair (6 ways) → lethal
- 2 symmetric translocations (no sticky ends) and 1 repair (6 ways) → viable

Half of the outcomes are lethal and half are viable, giving $P_{surv(mr)} = 0.5$, which the formula gives for $N_{mr} = 1$.

Now consider if 2 misrejoinings were simulated explicitly. If the second misrejoining was between the same pair of breaks as the first, then the situation was described above, with $P_{surv(mr)} = 0.5$. However, because the second misrejoining was between the same pair of breaks as the first, the second misrejoining was not counted toward N_{mr} (assumption 3), so $N_{mr} = 1$ and the formula correctly gives $P_{surv(mr)} = 0.5$.

If the second misrejoining was not between the same pair of breaks as the first ($N_{mr} = 2$), then the options are (after the third misrejoining; by assumption 2):

- 1 dicentric (no sticky ends), 1 symmetric translocation (no sticky ends) and 1 acentric fragment (no sticky ends) (36 ways) → lethal
- 3 symmetric translocations (no sticky ends) (12 ways) → viable

There are 36 ways for a lethal outcome and 12 ways for a viable outcome, giving $P_{surv(mr)} = 0.25$, which the formula correctly gives for $N_{mr} = 2$.

If the 3 misrejoinings were simulated explicitly, the situation is as described above, with $P_{surv(mr)} = 0.25$. In the current algorithm, the third misrejoining was mistakenly counted ($N_{mr} = 3$), so the formula incorrectly gave $P_{surv(mr)} = 0.125$. This will be fixed in future iterations of the algorithm.

Independent groups of breaks in a nucleus

Consider a nucleus containing one group of breaks with $N_{mr,1}$ and corresponding $P_{surv(mr),1}$ and a second group of breaks with $N_{mr,2}$ and corresponding $P_{surv(mr),2}$:

$$P_{surv(mr),1} = 0.5^{N_{mr,1}} \quad (1)$$

$$P_{surv(mr),2} = 0.5^{N_{mr,2}} \quad (2)$$

For the cell to be viable, both groups of breaks must have a viable outcome:

$$\begin{aligned} P_{surv(mr)} &= P_{surv(mr),1} P_{surv(mr),2} && (3)\\ &= 0.5^{N_{mr,1}} 0.5^{N_{mr,2}} && (4)\\ &= 0.5^{N_{mr,1}+N_{mr,2}} && (5) \end{aligned}$$

So $P_{surv(mr)}$ is given by the same formula but using the total number of misrejoinings in the nucleus.
